# Two New β-Dihydroagarofuran Sesquiterpenes from *Celastrus orbiculatus* Thunb and Their Anti-Proliferative Activity

**DOI:** 10.3390/molecules22060948

**Published:** 2017-06-09

**Authors:** Jingjing Zhou, Na Han, Guanghui Lv, Lina Jia, Zhihui Liu, Jun Yin

**Affiliations:** 1 Department of Pharmacognosy and Utilization Key Laboratory of Northeast Plant Materials, School of Traditional Chinese Medicine, Shenyang Pharmaceutical University, Shenyang 110016, China; zhoujingjing8338@163.com (J.Z.); hanna82@163.com (N.H.); lvguanghui09120@sina.com (G.L.); liuzhihuishenyang@163.com (Z.L.); 2 School of Life Science and Biopharmaceutics, Shenyang Pharmaceutical University, Shenyang 110016, China; frankjln@126.com

**Keywords:** *Celastrus orbiculatus* Thunb, β-dihydroagarofuran-type sesquiterpene, anti-proliferative

## Abstract

Two new β-dihydroagarofuran-type sesquiterpenes (**1**–**2**) were isolated and identified from the fruit of *Celastrus orbiculatus* Thunb, together with seventeen known compounds (**3**–**19**). The structures of the isolated new compounds were elucidated based on extensive spectroscopic analyses. The cytotoxic activities of the 19 sesquiterpenes on three cell lines, human acute promyelocytic leukemia HL-60, human leukemic K562, and human colon cancer HCT-116 cells, were evaluated in vitro*.* Compound **4** exhibited potent cytotoxic activity against HL-60, K562, and HCT116 cell lines with IC_50_ values of 3.61 μΜ, 17.13 μΜ and 10.15 μΜ, respectively, and the other compounds displayed moderate activity.

## 1. Introduction

*Celastrus orbiculatus* Thunb is a traditional herbal medicine used as a treatment for early tumors, and as a sedative and hypnotic [1,2]. *C. orbiculatus* possesses a broad range of bioactivities, which have attracted much interest, β-dihydroagarofuran-type sesquiterpenoids are characteristic natural products of Celastraceae and are regarded as important due to their biological activities, including cytotoxic [3], insecticidal [4], antitumor-promoting [5], anti-HIV [6], anti-inflammatory [7], immunosuppressant [8], and multidrug resistance (MDR) reversing activities [9]. Previous reports have shown that β-dihydroagarofuran sesquiterpenes isolated from Celastraceae species are effective anti-tumor compounds in vitro [10,11,12] and in vivo [13]. Additionally, β-dihydroagarofuran sesquiterpenes do not have any significant potential toxicity against normal human tumors in vivo [14], as shown in a previous study. In our own previous studies, the petroleum ether extracted fractions of *C. orbiculatus* exhibited significant cytotoxicity against human acute promyelocytic leukemia HL-60, human leukemic K562, and human colon cancer HCT-116 cells, was subjected to bioassay-guided fractionation.

In order to identify new bioactive compounds from the most effective fraction of *C. orbiculatus* with strong anti-proliferative activity, a detailed chemical investigation was carried out. In our present investigation, 2 new β-dihydroagarofuran-type sesquiteroenes (**1**–**2**) and 17 known compounds (**3**–**19**) were identified, together with their cytotoxic activity against human acute promyelocytic leukemia HL-60, human leukemic K562, and human colon cancer HCT-116 cells.

## 2. Results and Discussion

### 2.1. Two New Identified Compounds (**1**–**2**)

**Figure 1:**
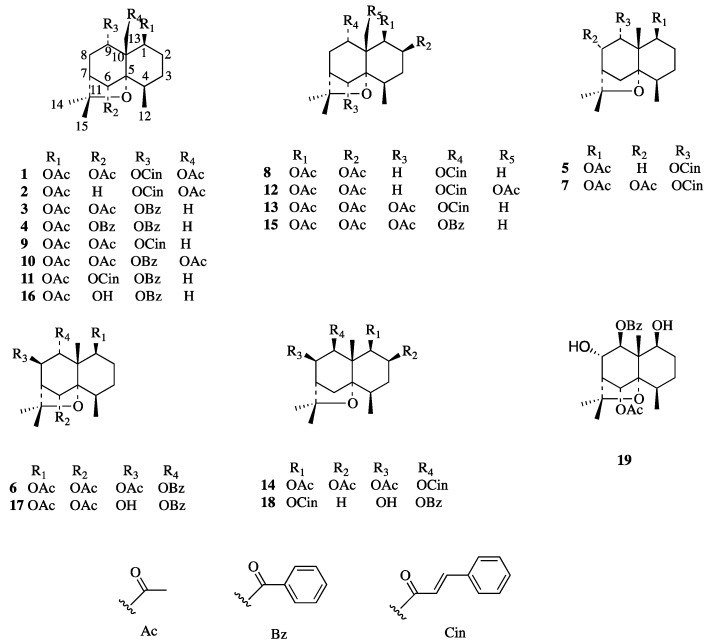
Structures of the β-dihydroagarofuran sesquiterpenes (**1**–**19**) assayed for their anti-proliferative activity.

Two new (**1**–**2**) and seventeen known (**3**–**19**) β-dihydroagarofuran-type sesquiterpenoids were isolated from the fruits of *Celastrus orbiculatus* Thunb using various chromatographic methods (Figure 1).

**Figure 2:**
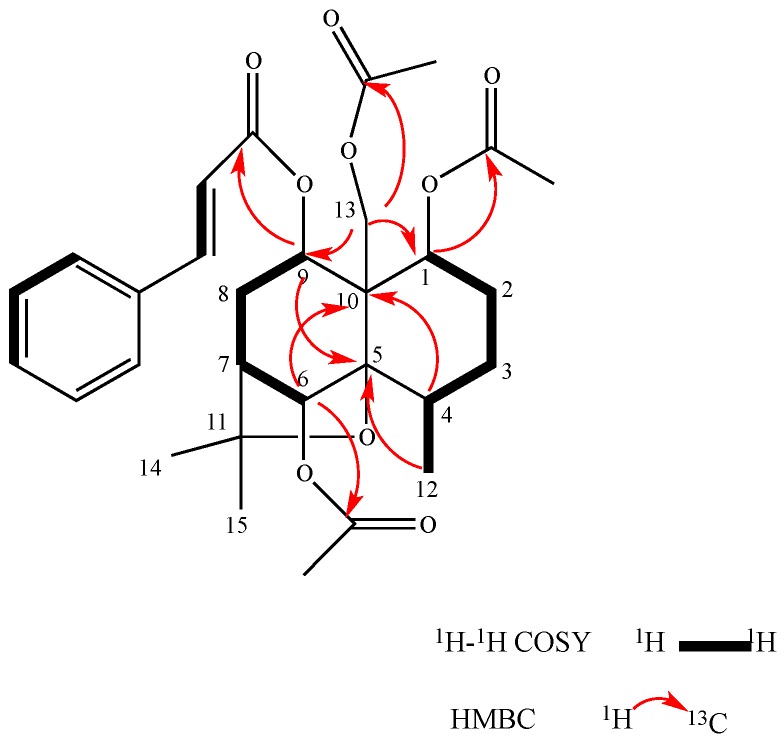
^1^H-^1^H COSY (bold) and selected HMBC (arrows) correlations of Compound **1**.

Compound **1** was obtained as a white amorphous powder and its molecular formula was found to be C_30_H_38_O_9_ by HRTOFMS (*m*/*z* 565.2461 [M + Na]^+^, calculated for 565.2460). Its IR spectrum showed absorption bands for ester group at 1725 and 1745 cm^−1^. The UV spectrum exhibited an absorption maximum at 230 and 270 nm, and the β-dihydroagarofuran skeleton was established from the ^1^H-^1^H COSY cross signals for the H-1/H-2/H-3/H-4/H-12 and H-6/H-7/H-8/H-9 spin systems and the HMBC correlations between H_2_-13 and both C-1 and C-9, between both H-9 and H_3_-12 and C-5, and between both H-4 and H-6 and C-10 (Figure 2). The ^13^C-NMR spectrum indicated that Compound **1** possesses a β-dihydroagarofuran skeleton based on 15 skeletal carbons, including δ_C_ 89.7 (C-5), 82.5 (C-11), 53.0 (C-10), and 48.8 (C-7), characteristic of a β-dihydroagarofuran skeleton. Its ^1^H-NMR spectrum showed signals for 7 aromatic protons for cinnamoyl groups at δ_H_ 6.36 (1H, d, *J* = 16.0 Hz), 7.68 (1H, d, *J* = 16.0 Hz), 7.54 (2H, m), and 7.38 (3H, m), three acetyl groups at δ_H_ 2.23 (3H, s), 2.09 (3H, s), and 1.80 (3H, s), three acylated oxymethine protons at δ_H_ 5.58 (1H, dd, *J* = 12.4 Hz, 4.5 Hz), δ_H_ 5.92 (1H, s), and δ_H_ 5.16 (1H, d, *J* = 7.3 Hz), and a pair of acylated oxymethine protons at δ_H_ 4.66 (1H, d, *J* = 12.2 Hz) and δ_H_ 4.41 (1H, d, *J* = 12.3 Hz), which indicated that Compound **1** is a four substituted β-dihydroagarofuran-type sesquiterpene, with three acetoxyl group and one cinnamoyl group. The assignments of the four substituent groups were determined based on the HMBC correlations between H-1 (δ_H_ 5.58) and the AcO-1 carbonyl carbon (δ_C_ 170.4), between H-6 (δ_H_ 5.92) and the AcO-6 carbonyl carbon (δ_C_ 170.2), between H-9 (δ_H_ 5.16) and the CinO-9 carbonyl carbon (δ_C_ 165.9), and between H_2_-13 (δ_H_ 4.66, δ_H_ 4.41) and the AcO-13 carbonyl carbon (δ_C_ 170.0), which indicated the locations of the four substituents of Compound **1** (Figure 2).

**Figure 3:**
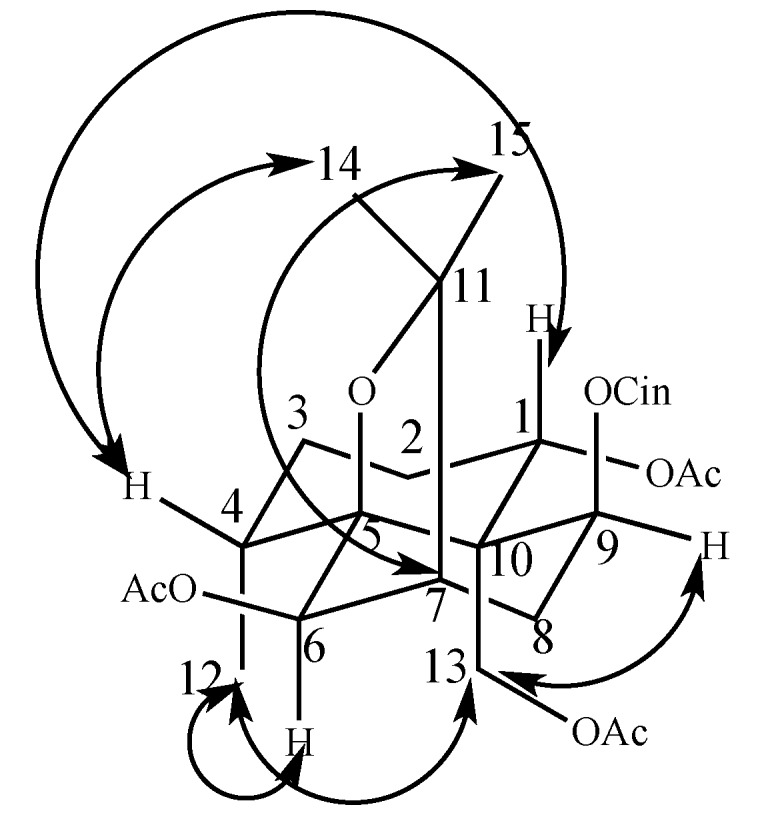
Key NOESY correlation of Compound **1**.

The relative configuration of Compound **1** was established by the coupling constants of the key proton signals and the NOESY spectrum. Generally, naturally occurring β-dihydroagarofuran sesquiterpenes exhibit β-orientations for H_2_-13 and H-7 [15,16]. The NOESY correlations of H_2_-13 and H_3_-12, H_2_-13 and H-9, H-6 and H_3_-12, and H_3_-15 and H-7 suggested that H_3_-12, H-9, H-6, and H-15 were in the β-orientation, while the α-orientation of H_3_-14 and H-1 were determined on the basis of NOESY correlations between H-4 and H_3_-14 and between H-1 and H-4 [17]. (Figure 3). Thus, Compound **1** was established as 1β,6α,13–triacetoxy-9α-cinnamoyloxy-β-dihydroagarofuran.

**Figure 4:**
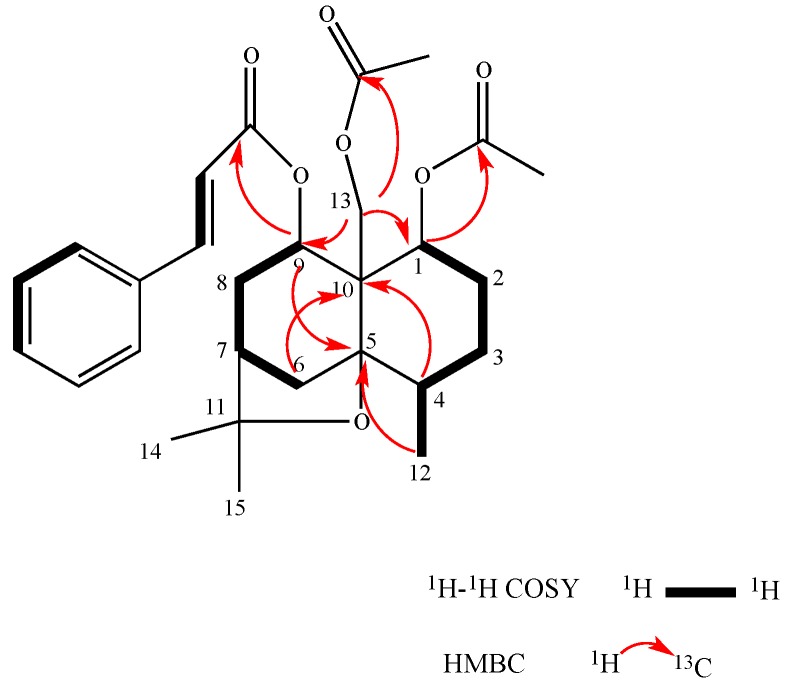
^1^H-^1^H COSY (bold) and selected HMBC (arrows) correlations of Compound **2**.

Compound **2** was isolated as colorless orthorhombic crystals. It had the molecular formula C_28_H_36_O_7_ as determined by HRTOFMS (*m*/*z* 507.2936 [M + Na]^+^, calculated for 507.2938). Its IR and UV spectrum were similar to those of Compound **1**. Based on a comparison of the NMR spectroscopic data of **2** with **1** (Table 1 and Table 2), it had the same β-dihydroagarofuran skeleton. One difference in the ^1^H-NMR spectrum of Compound **2** was the hydrogen group at C-6 compared with the acetate group in Compound **1**. Its ^1^H-NMR spectrum indicated signals for 7 protons in the aromatic region for cinnamoyl groups at δ_H_ 6.38 (1H, d, *J* = 16.0 Hz), 7.68 (1H, d, *J* = 16.0 Hz), 7.54 (2H, m), and 7.38 (3H, m), two acetyl groups at δ_H_ 2.16 (3H, s) and 1.80 (3H, s), two acylated oxymethine protons at δ_H_ 5.53(1H, dd, *J* = 12.5 Hz, 4.3 Hz) and δ_H_ 5.20 (1H, d, *J* = 7.0 Hz), and a pair of acylated oxymethine protons at δ_H_ 4.52 (1H, d, *J* = 12.1 Hz) and δ_H_ 4.48 (1H, d, *J* = 12.1Hz), which indicated that Compound **2** is a three-substituted β-dihydroagarofuran-type sesquiterpene, with two acetoxyl groups, and one cinnamoyl group. The assignments of the three substituent groups were determined based on the HMBC correlations between H-1 (δ_H_ 5.53) and the AcO-1 carbonyl carbon (δ_C_ 170.2), between H-9 (δ_H_ 5.20) and the CinO-9 carbonyl carbon (δ_C_ 166.2), and between H_2_-13 (δ_H_ 4.52, δ_H_ 4.48) and the AcO-13 carbonyl carbon (δ_C_ 170.8), which gave the locations of the three substituents of Compound **2** (Figure 4).

**Figure 5:**
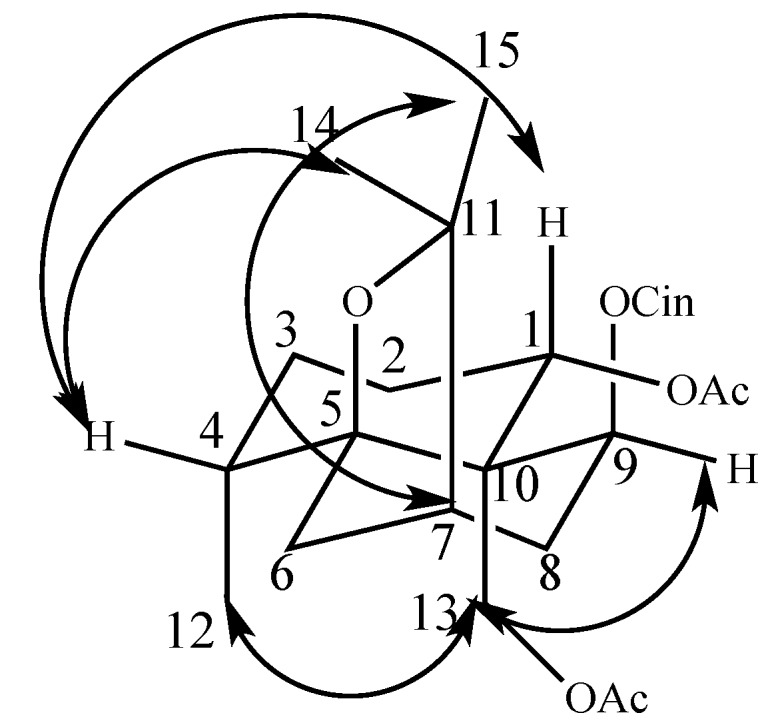
Key NOESY correlation of Compound **2**.

The relative configuration of Compound **2** was established by the coupling constants of the key proton signals and the NOESY data. The β-orientation of H-9 was determined on the basis of NOE correlations between H-9 and H_2_-13 (Figure 5) [18]. The NOESY correlations of H_2_-13 and H_3_-12, H_2_-13 and H-9, and H_3_-15 and H-7 suggested that H_3_-12, H-9, and H-15 had a β-orientation, while the α-orientation of H_3_-14 and H-1 were determined on the basis of NOESY correlations between H-4 and H_3_-14 and between H-1 and H-4. Thus, Compound **2** was determined as 1β,13–diacetoxy-9α-cinnamoyloxy-β-dihydroagarofuran.

### 2.2. Anti-Proliferative Activity of Compounds ***1**–**19*** on HL-60, K562, and HCT116 Cell Lines

In an initial study, the anti-proliferative activity of Compounds **1**–**19** on HL-60, K562, and HCT116 cell lines at 100 μM was tested by the MTT assay, with 5-FU as a positive control. It was shown that most of the compounds displayed more sensitive anti-proliferative activity on HL-60 and K562 than on HCT116. The compounds with a growth inhibition rate above 50% were chosen for further evaluation against the three kinds of tumor cell lines at a series of different concentrations to obtain the IC_50_ values (Table 3). Compounds **4** (IC_50_ values of 3.61 μΜ, 17.13 μΜ and 10.15 μΜ, respectively) showed stronger anti-proliferative activities on the three kinds of tumor cell lines than 5-FU. The other compounds exhibited selective potent activity toward the three kinds of tumor cell lines, such as Compound **13** (the growth inhibition rates for HL-60 and K562 at 100 μM were 56.29% and 55.14%, while the growth inhibition rates for HCT116 at 100 μM were 36.64%, respectively), while Compounds **9** and **14** (with growth inhibition rates on HCT116 at 100 μM of 70.42% and 77.89%, respectively) only had anti-proliferative activity on HCT116, which indicated that the cinnamoyl group at C-9 play an important role in the activity against HCT116 cells.

According to our research, β-dihydroagarofuran sesquiterpenes exhibit stronger the anti-proliferative activities against human acute promyelocytic leukemia HL-60 cells, human leukemic K562 cells, and human colon cancer HCT-116 cells. Following the in vitro results, we will perform further the in vivo studies of Compound **4** against cancer cells and study the mechanism of action of β-dihydroagarofuran sesquiterpenes, to investigate in more detail the potential cytotoxic activity of this series of sesquiterpenes.

## 3. Experimental

### 3.1. General Experimental Procedures

Optical rotations were recorded using KBr disks on a Perkin-Elmer 241MC polarimeter at room temperature. UV spectra were measured on a Shimadzu UV-2201 spectrophotometer. The IR spectra were recorded using a Bruker IFS-55 infrared spectrometer. NMR experiments were performed on Bruker-ARX-400 and Bruker-ARX-600 spectrometers in CDCl_3_, with tetramethylsilane (TMS) as an internal standard. The HRESIMS were obtained using a Brucker micro-TOF mass spectrometer, equipped with an ESI ion source operated in the positive-ion mode. Column chromatography (C.C) was performed on silica gel (100–200 mesh and 200–300 mesh, Qingdao Marine Chemical Co., Ltd. Qingdao, China) and Sephadex LH-20 columns (GE Healthcare, Uppsala, Sweden). Preparative HPLC was performed on a Welch ultimate XB-C18 column (250 × 10 mm, 5 μm) equipped with a pump and a single-wavelength UV detector. Analytical HPLC was conducted on a Shimadzu LC-10AVP UV-vis detector (Shimadzu Co., Ltd., Kyoto, Japan), and an N-2000 chromatographic work station (Intelligent Information Engineering Co., Ltd., Kyoto, Japan) using a C_18_ column (250 mm × 4.6 mm). TLC analysis was performed on silica-gel plates (Sil G/GF-254, Qingdao Marine Chemical Inc., Qingdao, China). 5-FU was obtained from Sigma Co., Ltd., Shanghai, China and had a purity of 99%. All chemical reagents used were obtained from Laibo Chemical Industries, Ltd. (Shenyang, China).

### 3.2. Plant Material

The fruits of *C. orbiculatus* were collected from the Mountain Hu′ er in the Liaoning province of China in September 2013 and were authenticated by Professor Jun Yin. A voucher specimen (ZJJ-NSTG-20130910) was deposited in the Herbarium of the Materia Medica, Department of Pharmacognosy, School of Traditional Chinese Materia Medica, Shenyang Pharmaceutical University, Shenyang, China.

### 3.3. Extraction and Isolation

The fruits of *C. orbiculatus* (5.0 kg) were extracted with EtOH–H_2_O (75:25, *v*/*v*) (50 L × 3), by refluxing for 2 h each time. The combined extracts were concentrated under reduced pressure to obtain a crude extract (700 g), which was dissolved in water and successively partitioned with petroleum ether (PE) (2 L × 5), EtOAc (2 L × 5), and n-butanol (2 L × 5). The PE extract was fractionated on a silica gel (200–300 mesh) column and eluted with a petroleum ether/EtOAc gradient (50:1, 30:1, 20:1, 10:1, 5:1, 1:1 *v*/*v*) to afford Fractions 1–5. Fr.1 (1 g) was further separated by column chromatography over silica-gel (200–300 mesh) with petroleum ether–acetone (100:1, 80:1, 50:1) to yield three subfractions. Subfraction 1.1 (200 mg) underwent silica-gel C.C with PE:acetone (80:1) to yield Compound **5** (20 mg), while Subfraction 1.2 (300 mg) was subjected to silica-gel C.C and eluted with petroleum ether–acetone (80:1, 60:1) to obtain Compounds **3** (15 mg) and **4** (30 mg). Subfraction 1.3 (40 mg) was recrystallized to yield Compound **6** (12 mg). Fr.2 (2.5 g) was subjected to silica-gel C.C (200–300 mesh, 50 g) and successively eluted with PE–acetone (50:1, 40:1, 30:1, *v*/*v*) followed by semipreparative HPLC to obtain Compounds **7** (10 mg), **8** (13 mg), **9** (20 mg), **10** (8 mg). Fr.3 (400 mg) was separated on a Sephadex LH-20 column with CH_2_Cl_2_–CH_3_OH (1:1) to obtain Compound **11** (18 mg) and Fr.3.1, and Fr.3.1 (240 mg) was then further separated by semipreparative HPLC (acetonitrile/water, 75:25, *v*/*v*) to obtain the new Compound **1** (10 mg) and the known Compound **13** (8 mg). Fr.4 (1.5 g) was further purified by silica-gel CC (200–300 mesh, 45 g) and successively eluted with PE–ethyl acetate (35:1, 30:1, 20:1, 10:1, *v*/*v*) to yield two subfractions 4.1–4.2. Then, Fr.4.1 (40 mg) was re-crystallized to yield Compound **12** (16 mg). Fr.4.2 (268 mg) was subjected by semipreparative HPLC (MeOH/water, 78:22, *v*/*v*) to obtain the new Compound **2** (6 mg) and the known Compounds **14** (20 mg) and **15** (14 mg). Fr.5. was subjected to a silica-gel CC with PE–acetone (20:1, 10:1, 5:1, 3:1) as eluents to obtain subfractions 5.1–5.2. Fr.5.1 (125 mg) was subjected to silica gel CC with PE–acetone (15:1) followed by re-crystallized to obtain Compounds **16** (15.5 mg) and **17** (9 mg). Fr.5.2 was subjected to reversed-phase HPLC using a 10 mm × 250 mm column, with MeOH:H_2_O (77:23) as eluent to give Compounds **18** (20 mg) and **19** (10 mg).

*β,6α,13-Triacetoxy-9α-cinnamoyloxy-β-dihydroagarofuran* (**1**). Colorless orthorhombic crystals; ${\left[\alpha \right]}_{D}^{20}$ 124.0 (MeOH); IR (KBr) γ_max_ 2925, 1748, 1721, 1605, 1459, 1369, 1236, 1099, 1034, 721 cm^−1^; UV (CH_2_Cl_2_) λ_max_ 230, 271. ^1^H-NMR (600 MHz, CDCl_3_): 5.58 (1H, dd, *J* = 12.4, 4.5 Hz, H-1), 5.92 (1H, s, H-6), 5.16 (1H, d, *J* = 7.3 Hz, H-9), 4.66 (1H, d, *J* = 12.2 Hz, H-13), 4.41 (1H, d, *J* = 12.3 Hz, H-13), 0.97 (3H, d, *J* = 7.4 Hz, H-12), 1.43 (3H, s, H-14), 1.39 (3H, s, H-15), 1.46–2.43 (8H, m, H-2, H-3, H-4, H-7, H-8). AcO [2.23 (3H, s), 2.09 (3H, s), 1.80 (3H, s)], CinO [6.36 (1H, d, *J* = 16.0 Hz), 7.68 (1H, d, *J* = 16.0 Hz), 7.54 (2H, m) 7.38 (3H, m)] .^13^C-NMR (150 MHz, CDCl_3_): 73.1 (C-1), 22.5 (C-2), 26.5 (C-3), 33.5 (C-4), 89.7 (C-5), 78.2 (C-6), 48.8 (C-7), 34.7 (C-8), 69.8 (C-9), 53.0 (C-10), 82.5 (C-11), 16.6 (C-12), 65.5 (C-13), 25.9 (C-14), 30.5 (C-15). AcO (170.7, 170.2, 170.0, 21.5, 21.3, 21.3), CinO (165.9, 145.8, 134.4, 130.6, 129.0, 128.4, 117.9); HR-ESIMS *m*/*z* 565.2461 [M + Na]^+^ (calculated for C_30_H_38_O_9_Na, 565.2460).

*1β,13-Diacetoxy-9α-cinnamoyloxy-β-dihydroagarofuran* (**2**). Colorless crystals; ${\left[\alpha \right]}_{D}^{20}$ 160 (MeOH); IR (KBr) γ_max_ 2930, 1748, 1721, 1625, 1450, 1364, 1236, 1079, 1024, 721 cm^−1^; UV (CH_2_Cl_2_) λ_max_ 232, 270.^1^H-NMR (600 MHz, CDCl_3_): 5.52 (1H, dd, *J* = 12.4, 4.5 Hz, H-1), 5.20 (1H, d, *J* = 6.8 Hz, H-9), 4.52 (1H, d, *J* = 12.2 Hz, H-13), 4.47 (1H, d, *J* = 12.3 Hz, H-13), 1.08 (3H, d, *J* = 7.4 Hz, H-12), 1.39 (3H, s, H-14), 1.19 (3H, s, H-15), 1.45–2.26 (10H, m, H-2, H-3, H-4, H-6, H-7, H-8), AcO [2.16 (3H, s), 1.80 (3H, s)], CinO [6.38 (1H, d, *J* = 16.0 Hz), 7.68 (1H, d, *J* = 16.0 Hz), 7.54 (2H, m) 7.38 (3H, m)]. ^13^C-NMR (150MHz, CDCl_3_): 73.4 (C-1), 22.8 (C-2), 26.8 (C-3), 40.1 (C-4), 87.0 (C-5), 36.7 (C-6), 43.6 (C-7), 33.9 (C-8), 69.9 (C-9), 50.6 (C-10), 82.0 (C-11), 17.4 (C-12), 65.0 (C-13), 24.4 (C-14), 30.3 (C-15). AcO (170.8, 170.2, 21.5, 21.3), CinO (166.2, 145.4, 134.6, 130.4, 129.0, 128.4, 118.3). HR-ESIMS *m*/*z* 507.2936 [M + Na]^+^ (calculated for C_28_H_36_O_7_Na, 507.2938).

### 3.4. Seventeen Known Compounds

1β,6α-diacetoxy-9α-benzoyloxy-β-dihydroagarofuran (**3**) [19], 1β-acetoxy-6α,9α-dibenzoyloxy-β-dihydroagarofuran (**4**) [19], 1β-acetoxy-9α-cinnamoyloxy-β-dihydroagarofuran (**5**) [20], 1β,6α,8β-triacetoxy-9α-benzoyloxy-β-dihydroagarofuran (**6**) [19], 1β,8α-diacetoxy-9α-cinnamoyloxy-β-dihydroagarofuran (**7**) [21], 1β,2β-diacetoxy-9α-cinnamoyloxy-β-dihydroagarofuran (**8**) [22], 1β,6α-diacetoxy-9α-cinnamoyloxy-β-dihydroagarofuran (**9**) [20], 1β,6α,13-triacetoxy-9α-benzoyloxy-β-dihydroagarofuran (**10**) [15], 1β-acetoxy-6α-cinnamoyloxy-9α-benzoyloxy-β-dihydroagarofuran (**11**) [23], 1β,2β,13-triacetoxy-9α-cinnamoyloxy-β-dihydroagarofuran (**12**) [24], 1β,2β,6α-triacetoxy-9α-cinnamoyloxy-β-dihydroagarofuran (**13**) [24], 1β,2β,6β-triacetoxy-9β-cinnamoyloxy-β-dihydroagarofuran (**14**) [25], 1β,2β,6α-triacetoxy-9α-benzoyloxy-β-dihydroagarofuran (**15**) [26], 1β-acetoxy-6α-hydroxydihydro-9α-benzoyloxy-β-dihydroagarofuran (**16**) [23]. 1β,6α-diacetoxy-8β-hydroxydihydro-9α-benzoyloxy-β-dihydroagarofuran (**17**) [27], 1β-cinnamyloxy-6α-acetoxy-8β-hydroxydihydro-9β-benzoyloxy-β-agarofuran (**18**) [23], 1β,8β-dihydroxy-6α-acetoxy-9β-benzoyloxy-β-dihydroagarofuran (**19**) [23] were isolated from the fruit of C*. orbiculatus*. The purities of these compounds were all above 98%. 5-FU [Sigma-Aldrich, Shanghai, China, 99% (purity)] was used as the positive control. All chemical regents used in this research were obtained from Laibo Chemical Company, Ltd., Shenyang, China.

### 3.5. Cell Culture

HL-60, K526, and HCT116 cell lines were used in this research. The three cell lines were purchased from American Type Culture Collection (ATCC, Rockville, MD, USA). HL-60, K526 and HCT116 were cultured in RPMI-1640 supplemented with 10% fetal bovine serum (FBS) and incubated at 37 °C in an atmosphere of 5% CO_2_ and 95% air. Stock solutions of the compounds for anti-proliferative assay were prepared in DMSO at an initial concentration of 50 or 100 mM.

### 3.6. In Vitro Anti-Proliferative Bioassay

The effect of Compounds **1**–**19** on cell proliferation was assessed by 3-(4,5-dimethylibiazol-2-yl)-2,5-diphenyl-tetrazolium bromide (MTT) assay [28]. Briefly, cells were seeded into 96-well microtiter plates at a density of 100 μL/well and incubated for 24 h. Culture media containing difference concentrations of the test samples were then added. After incubation for 72 h. One hundred microliters of MTT from a stock solution (0.5 mg/mL) was added to each well, and the plates were incubated for 4 h at 37 °C. The purple formazan produced was resuspended in 100 μL of DMSO using a multichannel pipette. The absorbance of the resulting formazan product was measured at 492 nm using a microplate reader (Tecan, Mnnedorf, Switzerland). All experiments were performed in triplicate. The percentage cell growth inhibition was calculated as follows:

Cell growth inhibition (%) = [OD_492_(control) − OD_492_(compound)]/OD_492_(control) × 100.

The IC_50_ values of the compounds inhibiting cell viability over 50% at a concentration of 100 μM were calculated. All cytotoxic activity data were analyzed by SPSS (20.0) and expressed as mean ± S.D.

## 4. Conclusions

In summary, the chemical constituents of the anti-proliferative fraction were investigated and 19 β-dihydroagarofuran sesquiterpenes were obtained, including two new compounds (**1**–**2**) and seventeen known compounds isolated from the fruit of *C. orbiculatus.* The structures of the two new compounds were characterized by an extensive analysis of 1D and 2D NMR and HRESIMS data, which is reported for the first time. In addition, Compound **4** exhibited a stronger toxic effect than 5-FU, most of these compounds exhibited moderate effects against HL-60, K562, and HCT116 cells as shown by an MTT assay. This showed that β-dihydroagarofuran sesquiterpenes are an important series of candidate compounds for anti-cancer drug research.

## Figures and Tables

**Table 1:** ^1^H-NMR Spectroscopic Data of **1** and **2** (600 MHz, CDCl_3_) ^a^.

Position	1	2
1	5.58 (1H, dd, *J* = 12.4 Hz, 4.5 Hz)	5.53 (1H, dd, *J* = 12.4 Hz, 4.3 Hz)
2	1.86 (1H, m), 1.55 (1H, m)	1.85 (1H, m), 1.55 (1H, m)
3	2.25 (1H, m), 1.46 (1H, m)	2.26 (1H, m), 1.45 (1H, m)
4	2.29 (1H, m)	1.86 (1H, m)
5		
6	5.92 (1H, s)	2.21 (1H, m), 2.01 (1H, m)
7	2.19 (1H, m)	2.04 (1H, m)
8	2.43 (1H, m), 2.15 (1H, m)	2.19 (1H, m), 2.07 (1H, m)
9	5.16 (1H, d, *J* = 7.3 Hz)	5.20 (1H, d, *J* = 6.8 Hz)
10		
11		
12	0.97 (3H, d, *J* = 7.4 Hz)	1.08 (3H, d, *J* = 7.8 Hz)
13	4.66 (1H, d, *J* = 12.2 Hz) 4.41 (1H, d, *J* = 12.3 Hz)	4.52 (1H, d, *J* = 12.2 Hz) 4.47 (1H, d, *J* = 12.1 Hz)
14	1.43 (3H, s)	1.39 (3H, s)
15	1.39 (3H, s)	1.19 (3H, s)

^a^ Data for additional ester groups are provided in the Experimental Section.

**Table 2:** ^13^C-NMR Spectroscopic Data of **1** and **2** (150 MHz, CDCl_3_) ^a^.

Position	1	2
1	73.1	73.4
2	22.5	22.8
3	26.5	26.8
4	33.5	40.1
5	89.7	87.0
6	78.2	36.7
7	48.8	43.6
8	34.7	33.9
9	69.8	69.9
10	53.0	50.6
11	82.5	82.0
12	16.6	17.4
13	65.5	65.0
14	25.9	24.4
15	30.5	30.3

^a^ Data for additional ester groups are provided in the Experimental Section.

**Table 3:** IC_50_ of Compounds **1**–**19** on three cancer cell lines.

Compounds	IC_50_ (μΜ)		
	HL-60	K562	HCT-116
**1**	-	-	-
**2**	23.11	35.00	50.64
**3**	26.34	38.75	46.61
**4**	3.61	17.13	10.15
**5**	33.29	42.85	34.25
**6**	19.32	37.27	37.46
**7**	30.85	30.20	-
**8**	-	-	-
**9**	-	-	36.03
**10**	20.32	35.84	41.64
**11**	-	-	-
**12**	-	-	-
**13**	40.50	42.67	-
**14**	-	-	30.98
**15**	31.51	40.86	50.01
**16**	33.51	42.56	-
**17**	18.87	35.22	-
**18**	-	-	-
**19**	23.55	47.31	31.05
**5-FU**	11.06	27.55	29.13

“-”: Not tested, IC_50_ values of compounds were calculated if they inhibited a tumor cell proliferation of over 50% at a concentration of 100 μΜ. Using 5-FU as a positive control.

## Supplementary Materials

^1^H-NMR, ^13^C-NMR, HMQC, HMBC, NOE, H-H COSY, HR-ESI-MS, and UV spectra for compound **1** and compound **2**, as well as the ^13^C-NMR data of 17 known compounds are available as Supporting Information. Supplementary data associated with this study can be found, in the online version.

## References

[1] Guo Y.Q. (2004). Research on the Chemical Constituents and Pharmacological Activities of *Celastrus orbiculatus* Thunb. Ph.D. Thesis.

[2] State Adiministration of Traditional Chinese Medicine (1999). Editorial Commission of Traditional Chinese Medicine.

[3] Carroll A.R., Davis R.A., Addepalli R., Fechner G.A., Guymer G.P., Forster P.I., Quinn R. (2009). Cytotoxic agarofurans from the seeds of the Australian rainforest vine *Celastrus subspicata*. J. Phytochem. Lett..

[4] Wu W.J., Wang M.G., Zhu J.B., Zhou W.M., Hu Z.N., Ji Z.Q. (2001). Five new insecticidal sesquiterpenoids from *Celastrus angulatus*. J. Nat. Prod..

[5] Takaishi Y., Ujita K., Tokuda H., Nishino H., Iwashima A., Fujita T. (1992). Errata. Cancer Lett..

[6] Horiuch M., Murakami C., Fukamiya N., Yu D., Chen T.H., Bastow K.F., Zhang D.C., Takaishi Y., Imakura Y., Lee K.H. (2006). Tripfordines A-C, Sesquiterpene Pyridine Alkaloids from Tripterygium wilfordii, and Structure Anti-HIV activity relationships of tripterygium alkaloids. J. Nat. Prod..

[7] Jin H.Z., Hwang B.Y., Kim H.S., Lee J.H., Kim Y.H., Lee J.J. (2002). Anti-inflammatory constituents of *Celastrus orbiculatus* inhibit the NF-γB activation and No production. J. Nat. Prod..

[8] Gutierrez-Nicolas F., Oberti J.C., Ravelo A.G., Estevez-Braun A. (2014). β-Agarofurans and sesquiterpene pyridine alkaloids from maytenus spinosa. J. Nat. Prod..

[9] Kennedy M.L., Cortes-Selva F., Perez-Victoria J.M., Jimenez I.A., Gonzalez A.G., Munoz O.M., Gamarro F., Castanys S., Ravelo A.G. (2001). Chemosensitization of a multidrug-resistant leishmania tropica line by new sesquiterpene from maytenus magellanica and maytenus chubutensis. J. Med. Chem..

[10] Jimenez I.A., Bazzocchi I.L., Nunez M.J., Mukainaka T., Tokuda H., Nishino H., Konoshima T., Ravelo A.G. (2003). Absolute configuration of sesquiterpenes from *Crossopetalum tonduzii* and their inhibitory effects on Epstein-Barr virus early antigen activation in Raji cells. J. Nat. Prod..

[11] Mendoza C.R., Jimenez I.A., Tokuda H., Kushida H., Bazzocchi I.L. (2005). Antitumor-promoting effects of new sesquiterpenes from *Crossopetalum tonduzii*. Chem. Biodivers..

[12] Gonzalez A.G., Tincusi B.M., Bazzocchi I.L., Tokuda H., Nishino H., Konoshima T., Jiménez I.A., Ravelo A.G. (2000). Anti-tumor rromoting effects of sesquiterpenes from Maytenus cuzcoina (Celastraceae). Bioorg. Med. Chem..

[13] Petrestelo N.R., Jimenez I.A., Tokuda H., Hayashi H., Bazzocchi I.L. (2010). Sesquiterpenes from *Maytenus jelskii* as potential cancer chemopreventive agents. J. Nat. Prod..

[14] Núñez M.J., Jiménez I.A., Mendoza C.R., Chavez-Sifontes M., Martinez M.L., Ichiishi E., Tokuda R., Tokuda H., Bazzocchi I.L. (2016). Dihydro-β-agarofuran sesquiterpenoids from celastraceae species as anti-tumor-promoting agents: Structure-activity relationship. Eur. J. Med. Chem..

[15] Tu Y.Q., Huang G.S., Ma Y.X., Wu X.L., Song Q.B. (1992). Alkaloids from *Celastrus angulatus*. J. Nat. Prod..

[16] Tu Y.Q., Hu Y.J. (1993). Structure of sesquiterpenoids from *Celastrus angulatus*. J. Nat. Prod..

[17] Wibowo M., Levrier C., Sadowski M.C., Nelson C.C., Wang Q., Holst J., healy P.C., Hofmann A., Davis R.A. (2016). Bioactive dihydro-β-agarofuran sesquiterpenoids from the Australian rainforest plant *Maytenus bilocularis*. J. Nat. Prod..

[18] Ning R., Lei Y., Liu S., Wang H., Zhang R., Wang W., Zhu Y., Zhang H., Zhao W. (2015). Natural β-dihydroagarofuran-Type Sesquiterpenoids as Cognition-Enhancing and Neuroprotective Agents from Medicinal Plants of the Genus *Celastrus*. J. Nat. Prod..

[19] Takaishi Y., Ohshima S., Nakano K., Tomimatsu T., Tokuda H., Nishino H., Iwashima A. (1993). Structures of Sesquiterpene Polyol Esters from *Celastrus stephanotiifolius* with Potential Tumor-Promotion Inhibitory Activity. J. Nat. Prod..

[20] Yoshihisa T., Shouji T., Kimiko N., Koutarou M., Toshiaki T. (1991). Structures of sesquiterpene polyol esters from *Tripterygium wilfordii* var. Regelii. Phytochemistry.

[21] Borrelli F., Borbone N., Capasso R., Montesano D., Angelo A.I., Simona D. M., Francesco C., Lydia F., Rocco L., Franco Z. (2004). New Sesquiterpenes with Intestinal Relaxant Effect from *Celastrus paniculatus*. Planta Med..

[22] Mingan W., Fuheng C. (1995). The study the insects antifeeding constituents of *Celastrus orbiculatus*. Chem. J. Chin. Univ..

[23] Zhu Y., Miao Z.J., Zhao W. (2008). Cytotoxic Dihydroagarofuranoid Sesquiterpenes from the Seeds of *Celastrus orbiculatus*. J. Nat. Prod..

[24] Guo Y.Q., Li X., Xu J., Meng D., Wang J. (2005). Sesquiterpene Esters from the Fruits *Celastrus orbiculatus*. Chem. Lett..

[25] Liu J.K., Becker H., Zapp J., Wu D. (1995). Four sesquiterpenes from the insecticidal plant *Celastrus angulatus*. Phytochemistry.

[26] Gao L., Zhang R., Lan J., Ning R., Wu D., Chen D., &Zhao W. (2016). β-Dihydroagarofuran-Type Sesquiterpenes from the Seeds of *Celastrus monospermus* and Their Lifespan-Extending Effects on the Nematode *Caenorhabditis elegans*. J. Nat. Prod..

[27] Tu Y., Chen Y., Wu D., Zhang X., Hao X. (1993). Sesquiterpenoids from *Celastrus paniculatus*. J. Nat. Prod..

[28] Rubinstein L.V., Shoemaker R.H., Paull K.D., Simon R.M., Tosini S., Skehan P., Scudiero D.A., Monks A., Boyd M.R. (1990). Comparison of in vitro anticancer-drug-screening data generated with a tetrazolium assay versus a protein assay against a diverse panel of human tumor cell lines. J. Natl. Cancer Inst..

