# Supplementary material for: Two New β-Dihydroagarofuran Sesquiterpenes from Celastrus orbiculatus Thunb and Their Anti-Proliferative Activity
Source: Molecules. 2017 Jun 9;22(6):948. doi: 10.3390/molecules22060948 (PMC6152708; doi:10.3390/molecules22060948)
Supplement: Supplementary file 1 [file molecules-22-00948-s001.docx]

^1^H-NMR spectrum of **1**

^13^C-NMR spectrum of **1**

HMQC spectrum of **1**

HMBC spectrum of **1**

^^

H^1^-H^1^ COSY spectrum of **1**

H^1^-H^1^ COSY spectrum of **1**

NOESY spectrum of **1**

NOESY spectrum of **1**


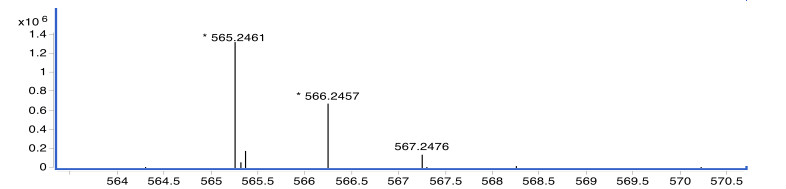


ESI MS of compound **1**

^1^H-NMR spectrum of **2**

^13^C-NMR spectrum of **2**

HMQC spectrum of **2**

HMBC spectrum of **2**

^1^H-^1^H COSY spectrum of **2**

^1^H-^1^H COSY spectrum of **2**

NOESY spectrum of **2**

NOESY spectrum of **2**


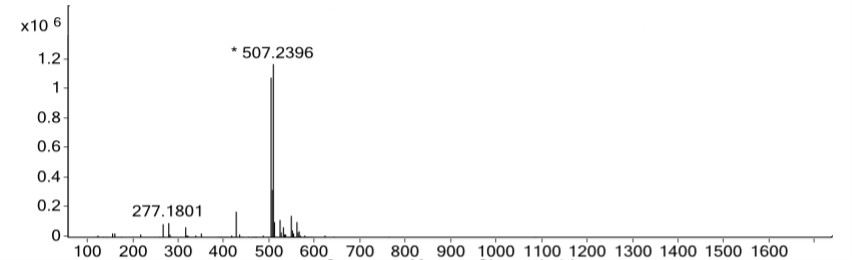


ESI MS of compound **2**
